# Metagenomic Nanopore Sequencing of Tickborne Pathogens, Mongolia

**DOI:** 10.3201/eid3014.240128

**Published:** 2024-11

**Authors:** Koray Ergunay, Bazartseren Boldbaatar, Brian P. Bourke, Laura Caicedo-Quiroga, Cynthia L. Tucker, Andrew G. Letizia, Nora G. Cleary, Abigail G. Lilak, Guugandaa Nyamdavaa, Sharav Tumenjargal, Michael E. von Fricken, Yvonne-Marie Linton

**Affiliations:** Walter Reed Biosystematics Unit, Smithsonian Institution Museum Support Center, Suitland, Maryland, USA (K. Ergunay, B.P. Bourke, L. Caicedo-Quiroga, C.L. Tucker, Y-M. Linton); Walter Reed Army Institute of Research, Silver Spring, Maryland, USA (K. Ergunay, B.P. Bourke, L. Caicedo-Quiroga, C.L. Tucker, Y-M. Linton); Smithsonian Institution National Museum of Natural History, Washington, DC, USA (K. Ergunay, B.P. Bourke, L. Caicedo-Quiroga, C.L. Tucker, Y-M. Linton); Mongolian University of Life Sciences School of Veterinary Medicine, Ulaanbaatar, Mongolia (B. Boldbaatar, G. Nyamdavaa, S. Tumenjargal); Naval Medical Research Unit INDO PACIFIC, Sembawang, Singapore (A.G. Letizia); University of Florida College of Public Health and Health Professions, Gainesville, Florida, USA (N.G. Cleary, A.G. Lilak, M.E. von Fricken)

**Keywords:** vector-borne infections, bacteria, parasites, viruses, tickborne illnesses, metagenomic sequencing, Mongolia

## Abstract

We performed nanopore-based metagenomic screening on 885 ticks collected from 6 locations in Mongolia and divided the results into 68 samples: 23 individual samples and 45 pools of 2–12 tick samples each. We detected bacterial and parasitic pathogens *Anaplasma ovis*, *Babesia microti, Coxiella burnetii*, *Borrelia miyamotoi*, *Francisella tularensis* subsp. *holarctica* and *novicida*, *Spiroplasma ixodetis*, *Theileria equi*, and *Rickettsia* spp., including *R. raoultii*, *R. slovaca*, and *R. canadensis*. We identified the viral pathogens Crimean-Congo hemorrhagic fever virus (2.9%), recently described Alongshan virus (ALSV) (2.9%), and Beiji nairovirus (5.8%). We assembled ALSV genomes, and maximum-likelihood analyses revealed clustering with viruses reported in humans and ticks from China. For ALSV, we identified surface glycoprotein markers associated with isolates from Asia viruses hosted by *Ixodes persulcatus* ticks. We also detected 20 virus species of unknown public health impact, including a near-complete Yanggou tick virus genome. Our findings demonstrate that nanopore sequencing can aid in detecting endemic and emerging tickborne pathogens.

A diverse spectrum of microbial agents, including viruses, bacteria, and protozoans, some with substantial health consequences or economic burden, can be transmitted to humans and animals by ticks (*1*). Surveillance plays a critical role in timely identification of tickborne pathogens and subsequent assessment of potential public health threats. Tickborne diseases constitute a major public and veterinary health threat in Mongolia, where a substantial portion of the population follows a pastoral lifestyle, including practices that involve frequent interactions with livestock animals and exposure to ticks (*2*). In this study, we used a combined pathogen screening strategy incorporating generic amplification and metagenomic sequencing in ticks collected from Mongolia. Research was conducted under an Institutional Animal Care and Use Committee–approved animal use protocol (protocol no. 21-01) in an American Association for Accreditation of Laboratory Animal Care International–accredited facility with a Public Health Services Animal Welfare Assurance and in compliance with the Animal Welfare Act and other federal statutes and regulations relating to laboratory animals. 

## Methods

We collected adult ticks by environmental dragging or removed ticks from sheep (*Ovis aries*) at 6 locations from the Bayan-Khongor, Selenge, and Umnugovi provinces in Mongolia during April 2021–May 2022. We morphologically identified, e-vouchered, and processed the tick samples as described elsewhere (*3*). We used nucleic acids from individual ticks for DNA barcoding and generic virus screening (*3*–*5*), then used the results to randomly assign each to a single or pooled sample (pool), in which we combined 5 μL of individual tick nucleic acids according to species and locality into 2–12 ticks per pool (Appendix 1 Figure 1). 

We performed nanopore-based metagenomic sequencing as described elsewhere (*3*). We performed similarity searches, de novo assembly, and read mapping on reads of >200 bp in Geneious Prime version 2022.2.1 (https://www.geneious.com). For mapping and pairwise comparisons, we used *Rickettsia* reference genomes (Appendix 2 Table 1). 

## Results

### Study Cohort and Pathogen Detection

We included 885 adult ticks in the study (Appendix 2 Table 1). We obtained uploaded DNA barcode sequences, specimen images, and collection data from information publicly available in the Barcode of Life Database (BOLD Systems, https://www.boldsystems.org) under project MONTK: Ticks of Mongolia (associated records MONTK001-23 through MONTK1128-23), where data are freely accessible. We separated 377 (42.6%) screened ticks into single-tick samples and 508 (57.4%) into multitick pools. We evaluated 68 samples, including 23 (33.8%) single-tick samples and 45 (66.2%) tick pools using nanopore-based metagenomic sequencing (Table 1; Appendix 1 Figure 1). We recovered no pathogens from *Hyalomma asiaticum* ticks from 2 (8.6%) single-tick samples or 6 (13.3%) pools. 

**Table 1:** Prevalence of microbial pathogens in b52 in a metagenomic nanopore sequencing study of tickborne pathogens, Mongolia*

Pathogens	*Dermacentor nuttalli *ticks			*Hyalomma asiaticum *ticks			*Ixodes persulcatus *ticks		Total no. (%)
	Single, n = 2	Pooled, n = 15		Single, n = 11	Pooled, n = 15		Single, n = 10	Pooled, n = 15	
Bacteria									
* Anaplasma ovis*	0	1		0	0		0	0	1 (1.4)
* Coxiella burnetii*	2	0		6	6		7	0	21 (30.0)
* Coxiella* spp. endosymbiont	0	7		0	0		0	0	7 (10.2)
* Borrelia miyamatoi*	0	0		0	1		0	0	1 (1.4)
* B. turcica*	0	0		0	0		0	1	1 (1.4)
* Francisella tularensis* subsp. *holarctica*	0	0		1	0		0	0	1 (1.4)
* F. tularensis* subsp. *novicida*	0	0		0	1		0	0	1 (1.4)
* F. persica* and *F. opportunistica*	0	0		10	12		0	0	22 (32.3)
* Rickettsia* spp. spotted fever group	1	15		3	2		10	15	46 (67.6)
* Rickettsia* spp. endosymbiont	0	7		1	0		8	15	31 (45.5)
* Spiroplasma ixodetis*	0	0		0	0		0	5	5 (7.3)
Viruses									
Alongshan virus	0	0		0	0		0	2	2 (2.9)
Beiji nairovirus	0	0		0	0		0	4	4 (5.8)
Crimean-Congo hemorrhagic fever virus	0	0		2	0		0	0	2 (2.9)
Parasites									
* Babesia microti*	0	0		2	0		1	1	4 (5.8)
* Theileria equi*	0	3		0	0		0	0	3 (4.4)

*Tick-associated opportunistic or endosymbiotic bacteria closely related to pathogenic species are provided for comparison.

### Bacteria and Protozoa 

Spotted fever group (SFG) *Rickettsia* and rickettsial endosymbionts were the most prevalent tickborne bacteria (Table 1). We identified the infecting species as *R. canadensis* in 5 single-tick samples (5/14, 35.7%) and 2 pools (2/32, 6.2%), *R. raoultii* in 8 pools (8/33, 24.2%), and *R. slovaca* in 1 single-tick sample (1/14, 7.1%). We detected *R. canadensis* and *R. slovaca* in *Ixodes persulcatus* ticks and *R. raoultii* in pools of *Dermacentor nuttalli* ticks and from 1 single-tick *Hy. asiaticum* sample (Appendix 2 Tables 2, 3). 

Assembly and maximum-likelihood analysis of the *R. canadensis* contig (GenBank accession no. PP158215) encompassing the *rplO*, *rpmD*, and *secY* regions from an *I. persulcatus* tick sample showed a differential grouping of the Mongolia sequence within the *R. canadensis* clade, distinct from the SFG (Appendix 1 Figure 2). Analysis of *mutS* and *uvrD* contigs from a *D. nuttalli* tick pool revealed similar clustering of those sequences with *R. raoultii* strains (Appendix 1 Figure 3). In the remaining samples, we could not identify specific species, presumably because multiple SFGs were present. In individual ticks, we documented coinfections with SFG and endosymbionts in 1 *Hy. asiaticum* and 8 *I. persulcatus* ticks. We assembled complete plasmid sequences of *R. raoultii* from 5 *D. nuttalli* tick pools, which revealed 94.3%–97.6% identity with *R. raoultii* strain Khabarovsk plasmid pRra3 (GenBank accession no. CP010972). 

We observed pathogenic and opportunistic *Francisella* species exclusively in *Hy. asiaticum* ticks. We detected *F. tularensis* subsp. *holarctica* in 1 single-tick sample and subsp. *novicida* in 1 tick pool, with an overall combined prevalence of 2.9% (2/68). Opportunistic species, including *F. persica* and *F. opportunistica*, were more common, identified in 32.3% of all samples, including those with pathogenic *Francisella* species. 

We detected *Coxiella burnetii* in all 3 tick species examined, with an overall prevalence of 30.8%. Unlike other endosymbionts, *Coxiella*-like bacteria were less commonly detected (10.2%) and only in *D. nutalli* ticks. We detected *Borrelia miyamotoi*, an agent of tickborne relapsing fever, in *Hy. asiaticum* ticks (1 pool); *Borrelia turcica* was detected only in *I. persulcatus* ticks*.* Other tickborne bacteria identified included *Spiroplasma ixodetis*, detected in 5 (7.3%) *I. persulcatus* tick pools, and *Anaplasma ovis*, detected in 1 (1.4%) pool of *D. nutalli* ticks. Among tickborne protozoan parasites, we detected *Babesia microti*, causative agent of human piroplasmosis, in 5.8% and *Theileria equi*, causative agent of equine piroplasmosis, in 4.4% of samples. We identified *B. microti* piroplasm, which also causes human babesiosis, in single-tick and pooled *Hy. asiaticum* and *I. persulcatus* tick samples; we detected *T. equi* only in *D. nutalli* tick pools (Table 1). 

### Viruses 

We detected 3 tickborne viruses of human health concern: Crimean-Congo hemorrhagic fever virus (CCHFV) (family Nairoviridae, *Orthonairovirus hemorrhagiae*), Beiji nairovirus (BJNV) (family Nairoviridae, *Norwavirus beijiense*), and Alongshan virus (ALSV) (unclassified species of family Flaviviridae) (Table 1) (*6*). After preliminary reactivity in generic virus screening, we detected CCHFV in only 2 (2.9%) individual *Hy. asiaticum* ticks. Available sequence information revealed reads of CCHFV small and medium genome segments and displayed high identities to CCHFV genomes from the Inner Mongolia Autonomous Region of China (Appendix 2 Table 2). Reads and contigs of BJNV large and small segments in 4 (5.8%) *I. persulcatus* tick pools showed high pairwise identities with isolates previously characterized elsewhere (Appendix 2 Table 3). Finally, we detected ALSV in 2 (2.9%) pools of *I. persulcatus* ticks. We were able to generate sequences of all ALSV genome segments from both pools (Appendix 2 Table 3), and assemble the coding regions in pool b52, tentatively designated ALSV-Mongolia-b52 (GenBank accession nos. PP125347–50). Phylogenic construction revealed clustering of individual segments with ALSV documented from the Inner Mongolia Autonomous Region and Heilongjiang Province in China (Appendix 1 Figures 4–7). Pairwise comparisons based on complete glycoprotein sequences encoded on segment 2 located the ALSV-Mongolia-b52 strain within the Asia subgroup of the *I. persulcatus* tick isolates (Table 2) (*7*). 

**Table 2:** Amino acid substitutions in the Alongshan virus VP1a, VP1b, and nuORF proteins compared with ALSV-Mongolia-b52 in a metagenomic nanopore sequencing study of tickborne pathogens, Mongolia*

Virus protein	Amino acid position	*Ixodes ricinus* tick group	*Ixodes persulcatus* tick group		ALSV-Mongolia-b52
			Europe subgroup	Asia subgroup	
VP1a	8	Ala	Ala	Thr	Thr
	72	Val	Ala	Ala	Ala
	115	Ala	Val	Val	Val
	135	Val	Lys	Lys	Lys
	138	Pro	Ser	Ser	Ser
	153	Lys	Arg	Arg	Arg
	210	Gly	Gly	Ser	Ser
	216	Thr	Ala	Ala	Ala
	321	Val	Val	Thr	Thr
	460	Thr	Met	Thr	Thr
	472	Arg	His	His	His
	476	Arg	Arg	Gln	Gly
VP1b	58	Met	Met	Leu	Leu
	112	Ile	Ile	Val	Val
	127	Lys	Lys	Arg	Arg
	135	Ser	Ser	Gly	Gly
	216	Val	Ile	Ile	Ile
nuORF	4	Lys	Lys	Gly	Gly
	15	Asp	Asp	Asn	Asn
	132	Thr	Ala	Thr	Thr

*ALSV, Alongshan virus; nuORF, novel upstream open reading frame; VP, virus capsid protein.

We identified sequences of 20 additional virus species belonging to 6 virus families, none of which are currently known to cause symptomatic disease in humans or animals (Appendix 1 Table 2). We recovered complete or near-complete coding sequences of Yanggou tick virus (unclassified species of family Flaviviridae) from 1 *D. nuttalli* tick pool (YGTV-Mongolia-b77; GenBank accession nos. PP125351–54), distinctly clustered with related viruses in the maximum-likelihood analysis (Appendix 1 Figures 4–7).

## Discussion

In this study, we used a metagenomic sequencing–based approach to detect and characterize tickborne pathogens agnostically in comparable numbers of single-tick and pooled tick samples, representing 3 tick species endemic to Mongolia. We detected pathogenic bacteria, viruses, or parasites in 86.7% of samples with a predominance of bacterial pathogens (Table 1). The most frequently observed bacteria were SFG *Rickettsia* (67.6%) and related endosymbionts (45.5%). Bacteria of the genus *Rickettsia*, gram-negative obligate intracellular bacteria, account for most bacterial infections transmitted by ticks (*8*). SFG includes >30 distinct *Rickettsia* species associated mainly with symptoms of spotted fever. Previous reports using various detection approaches have documented several *Rickettsia* species associated with spotted fever in Mongolia and the neighboring Inner Mongolia Autonomous Region of China (*2*). We identified *R. raoultii*, *R. slovaca*, and *R. canadensis* in single-tick and pooled tick samples from Mongolia. Initially reported in *Haemaphysalis leporispalustris* ticks from Canada, *R. canadensis* was also detected in *Haemaphysalis japonica* ticks from far eastern Russia, *Haemaphysalis flava* ticks from South Korea, and *Haemaphysalis longicornis* and *I. persulcatus* ticks from China. Monophyletic clustering based on *ompB* and *gltA* genes, observed in this study and elsewhere, suggests that *R. canadensis* constitutes an independent group (*9*). Although its pathogenicity is unclear, serologic evidence of *R. canadensis* exposure in humans has been documented (*10*), making the pathogen a potential agent of tickborne infections in endemic regions, including Mongolia. 

Other bacterial pathogens detected in ticks, including *A. ovis*, *C. burnetii*, and *B. miyamotoi*, have been documented previously in various regions in Mongolia (*2*). We further detected *F. tularensis* subsp. *holarctica*, a subspecies that causes human tularemia, and *F. tularensis* subsp. *novicida* in *Hy. asiaticum* ticks collected from different locations. In contrast to tularemia agents, *F. tularensis* subsp. *novicida* rarely causes human disease and, even then, mostly involving persons with coexisting medical conditions or immunosuppression (*11*). Cases of human tularemia have been reported in Mongolia; therefore, our findings documenting the presence of *Francisella* species bacteria associated with human infections helps define the epidemiology of this pathogen. Finally, we identified the intracellular mollicute *S. ixodetis* in pooled *I. persulcatus* tick samples in Mongolia. Although members of the genus *Spiroplasma* are vertically transmitted endosymbionts of Ixodid ticks, cases of acute febrile illness caused by *S. ixodetis* have been documented in both immunocompromised and immunocompetent adults with frequent tick exposure (*12*). In addition to those bacteria, we identified the Piroplasmorida apicomplexans parasites *B. microti* and *T. equi*, previously reported to circulate in various locations in Mongolia (*2*). Despite the presence of those and other potentially pathogenic species, no information on human infections is currently available. 

Tickborne viral pathogens detected in the study include CCHFV, BJNV, and ALSV in 11.7% (8/68) of samples. We identified sequences of CCHFV in low abundance in individual *Hy. asiaticum* tick samples (Appendix 2 Table 2). Previous screenings carried out in various regions have described a low frequency of CCHFV in *Hy. asiaticum* ticks (6). Of note, relatively high CCHFV seroprevalence in humans has been documented, as well as detectable antibodies in various mammals that might serve as reservoirs, although no human cases have been documented to date (*6*). BJNV has recently been included as a species in *Norwavirus* genus (family Nairoviridae), members of which lack the medium genome segment encoding for the structural glycoproteins in other nairoviruses, such as CCHFV (*6*). BJNV was described in tick-associated human febrile disease of unknown etiology from the Inner Mongolia Autonomous Region of China, further displaying pathogenicity in cell lines and experimental infections (*13*). Virus-specific antibodies were detected with a prevalence of up to 54.6% in human convalescent serum, as well as in sheep and cattle from the region. Detection of virus nucleic acids were reported from several tick species in China (*13*). BJNV was the most prevalent tickborne virus in our study at a 5.8% detection rate; we found the virus exclusively in *I. persulcatus* tick pools (Table 1). 

ALSV is another recently described virus with human health effects. It was originally described in in patients in the Inner Mongolia Autonomous Region and Heilongjiang Province of China with febrile diseases and a history of tick bites, followed by seroconversion (*14*). ALSV particles are enveloped, possessing positive-sense single-stranded RNA genomes in 4 segments and classified in the Jingmenvirus group of *Flaviviridae* because of nonstructural protein homologies. ALSV has been reported in various tick species collected in several countries in Eurasia and showed evidence of viral replication and exposure in sheep, cattle, and deer (*7**,**15*). We detected ALSV in 2 *I. persulcatus* tick pools and assembled the prototype virus genome (ALSV-Mongolia-b52). Maximum-likelihood analyses revealed grouping of ALSV-Mongolia-b52 with viruses reported from humans and ticks from China. Further analysis of the putative virus VP1a and VP1b surface glycoprotein sequences revealed amino acid markers associated with ASLV isolates from Asia hosted by *I. persulcatus* ticks (Table 2) (*7*). Those findings confirm the presence of ASLV in Mongolia and indicate *I. persulcatus* ticks are a probable vector. As with BJNV and many other tickborne infections, human ALSV infections are reported as tick bite–associated, nonspecific febrile illnesses. Diagnostic assays are imperative to determine the public health burden of those emergent human pathogens. 

Among limitations, the study cohort was composed of 3 tick species that represent frequently documented tickborne pathogen vectors, including those species mostly observed in the northern part of Mongolia that is covered by boreal forest (*I. persulcatus* ticks), in the northern and central steppes (*D. nuttalli* ticks), and in the southern aimag areas (*Hy. asiaticum* ticks) (*2*). Nevertheless, we have described several other tick species within the genera *Dermacentor*, *Haemaphysalis*, *Hyalomma*, *Ixodes*, and *Rhipicephalus*, although some have been observed only rarely and include scarce information on public health effects (*2*). Our screening strategy included molecular barcoding and generic virus screening for individual ticks, whereas we pooled randomly selected samples of identical tick species and collection site. We performed microbial characterization of prescreened single-tick and pooled tick samples using metagenomic sequencing. Despite random assignment of ticks for individual or pooled screening, target genome quantity and infection rates of particular microorganisms might further hamper detection and might not correlate with actual prevalence. Therefore, we presented only pathogen detection rates and did not calculate the maximum-likelihood estimates or minimum infection rates, which are frequently generated based on data from target-specific assays. Nevertheless, incorporating morphology and barcoding for accurate species identification and generic testing as needed, combined with metagenomic sequencing for accurate pathogen characterization is an efficient strategy. Because of limited sample availability, we could not perform a systematic comparison of single-tick versus pooled tick testing or pathogen detection rates between collection sites. However, our findings demonstrate the utility of a metagenomic sequencing approach for detecting and characterizing both endemic and emerging pathogens, especially for locations with limited pathogen information. 

In conclusion, we documented several bacterial and viral pathogens, some of which were initially described in ticks from Mongolia. Although the public health impact of those pathogens remains unclear, our findings demonstrate that nanopore sequencing can aid in detecting endemic and emerging tickborne pathogens.

Appendix 1 Additional information on distribution and tick species in a metagenomic nanopore sequencing study of tickborne pathogens, Mongolia.

Appendix 2 Additional information on *Rickettsia* reference genomes used in metagenomic nanopore sequencing of tickborne pathogens, Mongolia.
