# Supplementary material for: Metagenomic Nanopore Sequencing of Tickborne Pathogens, Mongolia
Source: Emerg Infect Dis. 2024 Nov;30(Suppl 2):S105–10. doi: 10.3201/eid3014.240128 (PMC11559569; doi:10.3201/eid3014.240128)
Supplement: Appendix 1 — Additional information on distribution and tick species in a metagenomic nanopore sequencing study of tickborne pathogens, Mongolia. [file 24-0128-Techapp-s1.pdf]

Article DOI: https://doi.org/10.3201/eid3014.240128

EID cannot ensure accessibility for supplementary materials supplied by authors. Readers who have difficulty accessing supplementary content should contact the authors for assistance.

Metagenomic Nanopore Sequencing of Tickborne Pathogens, Mongolia

Appendix 1

Appendix 1 Table 1. Distribution and species composition of tick and processing approach according to sampling sites. Where multiple samples were pooled, numbers of specimens are listed followed by numbers of pools given in parentheses.

	Dermacentor nuttalli		*Hyalomma asiaticum*		*Ixodes persulcatus*		
Location	Single	Pooled (no.)	Single	Pooled (no.)	Single	Pooled (no.)	Total no. (%)
Site 1 (Lat. 44.492, Long.105.542)	NA	NA	100	NA	NA	NA	100 (11.2)
Site 2 (Lat. 44.490, Long.105.531)	NA	NA	NA	176 (15)	NA	NA	176 (19.8)
Site 3 (Lat. 49.774, Long. 107.604)	NA	NA	NA	NA	188	156 (15)	344 (38.8)
Site 4 (Lat. 46.91, Long. 99.151)	89	36 (3)	NA	NA	NA	NA	125 (14.1)
Site 5 (Lat. 46.03, Long. 100.852)	NA	36 (3)	NA	NA	NA	NA	36 (4)
Site 6 (Lat. 48.415, Long. 90.986)	NA	104 (9)	NA	NA	NA	NA	104 (11.7)
Total count (%)	265 (29.9)		276 (31.1)		344 (38.8)		885
Total pools	15		15		15		45

*Lat., latitude; Long., longitude; NA, not applicable.

Appendix 1 Table 2. Prevalence of viruses with unknown health impact.

		Dermacentor nuttalli		*Hyalomma asiaticum*		*Ixodes persulcatus*		
Family	Virus	Single, n = 2	Pooled, n = 15	Single, n = 11	Pooled, n = 15	Single, n = 10	Pooled, n = 11	Total no. (%)
Chuviridae	Bole tick virus 3	NA	NA	NA	10	NA	NA	10 (14.7)
	Lesnoe mivirus	NA	NA	NA	NA	NA	1	1 (1.4)
Flaviviridae	Yanggou tick virus	NA	5	NA	NA	NA	NA	5 (7.3)
Hepeviridae	*Hepeviridae* sp.	NA	NA	NA	1	NA	NA	1 (1.4)
Nairoviridae	Gakugsa tick virus	NA	NA	NA	NA	2	NA	2 (2.9)
	Nairoviridae sp. isolate L1	NA	NA	NA	NA	NA	1	1 (1.4)
Phenuiviridae	Alxa tick phlebovirus	NA	NA	NA	1	NA	NA	1 (1.4)
	Blacklegged tick phlebovirus	NA	NA	NA	NA	1	NA	1 (1.4)
	Bole tick virus 1	NA	NA	11	NA	NA	NA	11 (16.1)
	Changping tick virus 1	NA	2	NA	NA	NA	NA	2 (2.9)
	Mukawa virus	NA	NA	NA	NA	NA	1	1 (1.4)
	Onega tick phlebovirus	NA	NA	NA	NA	NA	1	1 (1.4)
	Sara tick phlebovirus	NA	NA	NA	NA	NA	7	7 (10.2)
Rhabdoviridae	Taishun tick virus	NA	NA	NA	6	NA	NA	6 (8.8)
Unclassified	Bole *H. asiaticum* virus 1	NA	NA	NA	4	NA	NA	4 (5.8)
	Bole tick virus 4	NA	NA	NA	5	NA	NA	5 (7.3)
	Bunyavirales spp. isolate L1b	NA	NA	NA	NA	NA	1	1 (1.4)
	Hubei toti–like virus 24	NA	3	NA	NA	NA	NA	3 (4.4)
	I. scapularis–associated virus 1	NA	NA	NA	NA	NA	1	1 (1.4)
	Xinjiang tick–associated virus 1	NA	2	NA	NA	NA	NA	2 (2.9)

NA, not applicable.

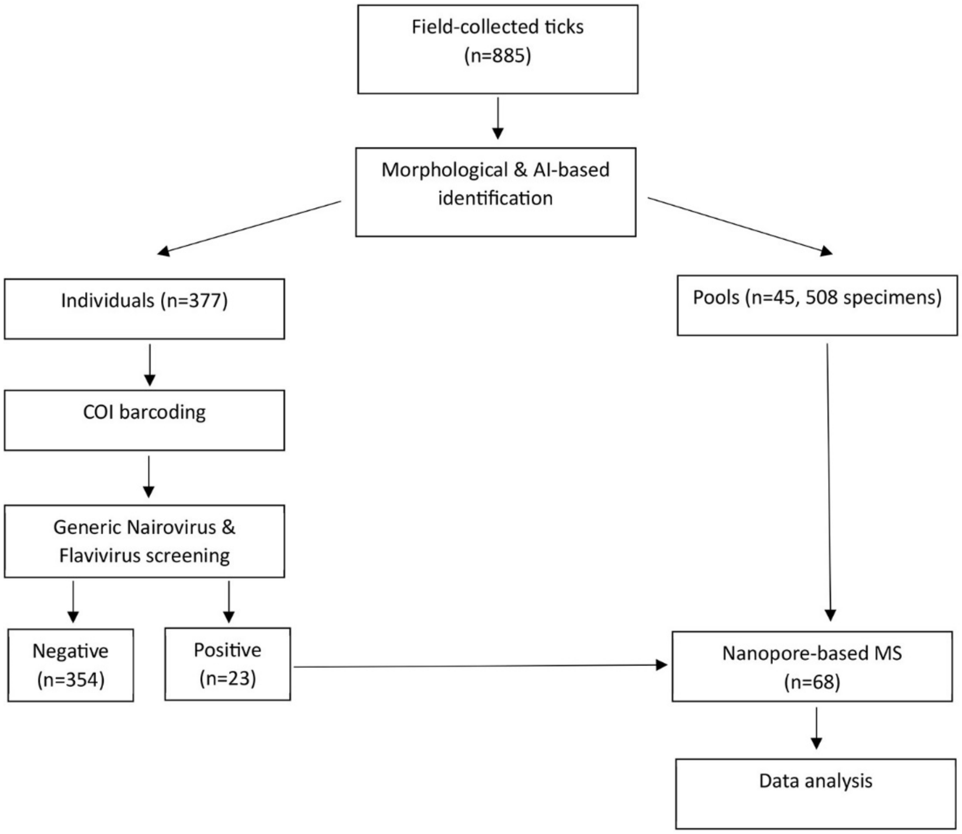

Appendix 1 Figure 1. Schema of the screening approach.

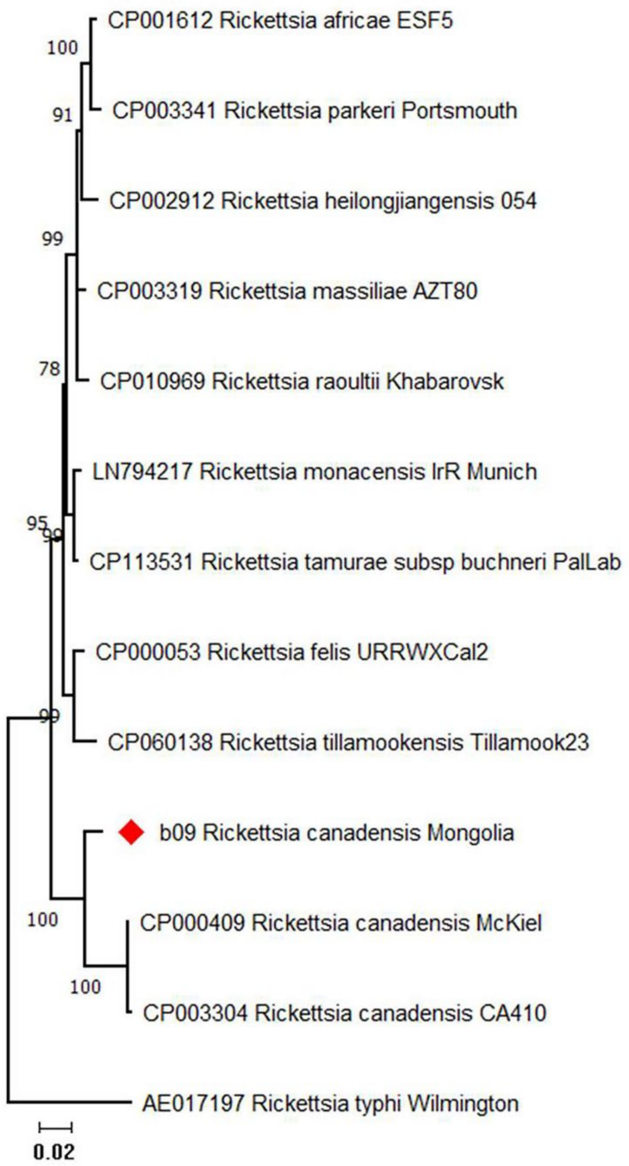

Appendix 1 Figure 2. Maximum-likelihood consensus tree of the partial *Rickettsia secY*, *rplO*, and *rpmD* genes (1,442 nucleotides), constructed using Tamura-Nei model, gamma distributed with invariant sites (G+I) for 500 replications. Red diamond indicates *Rickettsia canadensis* sequence obtained in the study and indicated with sample identifier. *Rickettsia* strains are indicated by GenBank accession number, name, and isolate identifier.

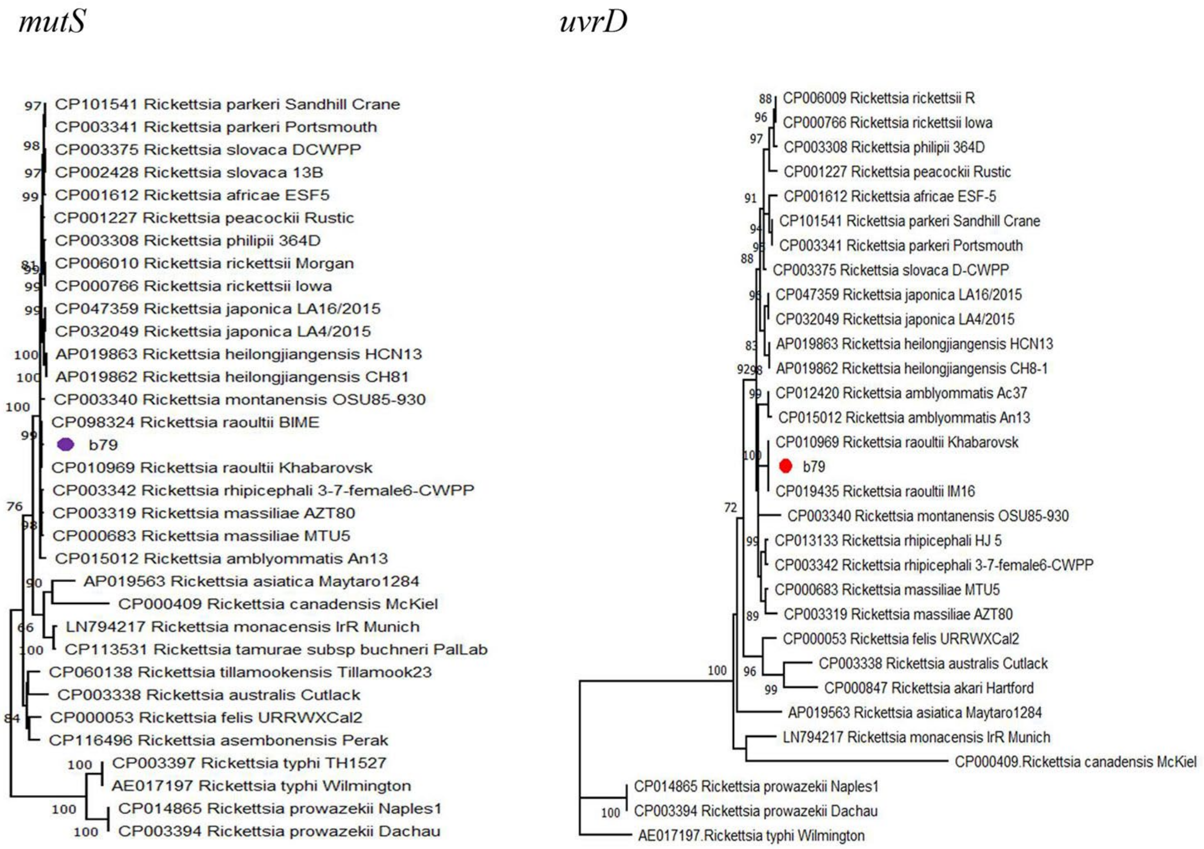

Appendix 1 Figure 3. Maximum-likelihood consensus tree of the *Rickettsia mutS* (2,649 nucleotides) and *uvrD* (1,080 nucleotides), constructed using Tamura-Nei model, gamma distributed with invariant sites (G+I) for 500 replications. Sequences obtained in the study are marked and indicated with sample identifiers. *Rickettsia* strains are indicated by GenBank accession number, name, and isolate identifier.

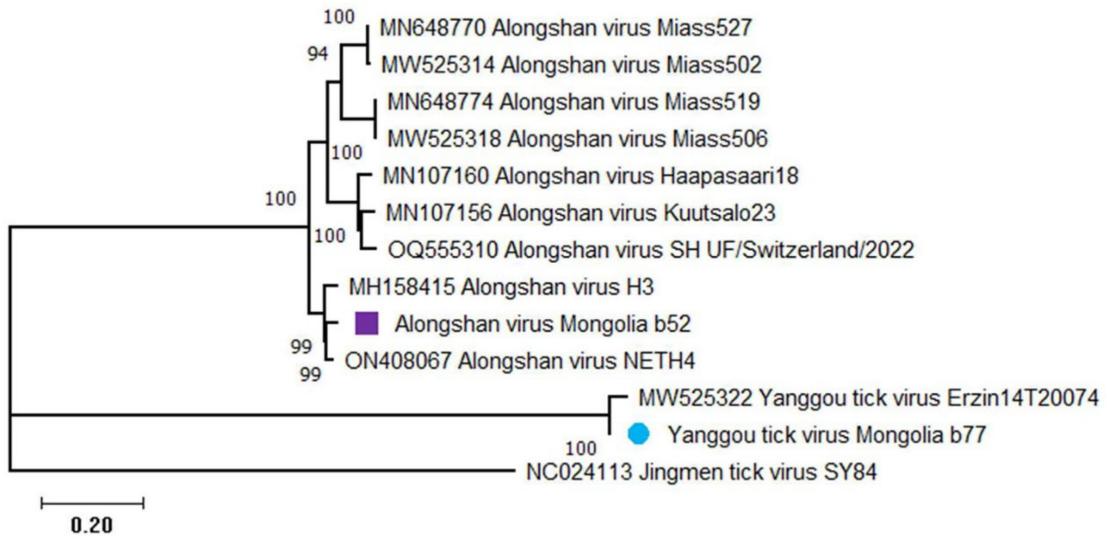

Appendix 1 Figure 4. Maximum-likelihood tree of the Alongshan virus and Yanggou tick virus NS5-like protein sequences (segment 1; 1,482 nucleotides), constructed using Tamura-Nei model, gamma distributed with invariant sites (G+I) for 500 replications. Sequences obtained in the study are marked (purple square and blue octagon) and indicated with sample identifiers. Virus strains are indicated by GenBank accession number, name and isolate identifier. Jingmen tick virus strain SY84 was included as an outgroup.

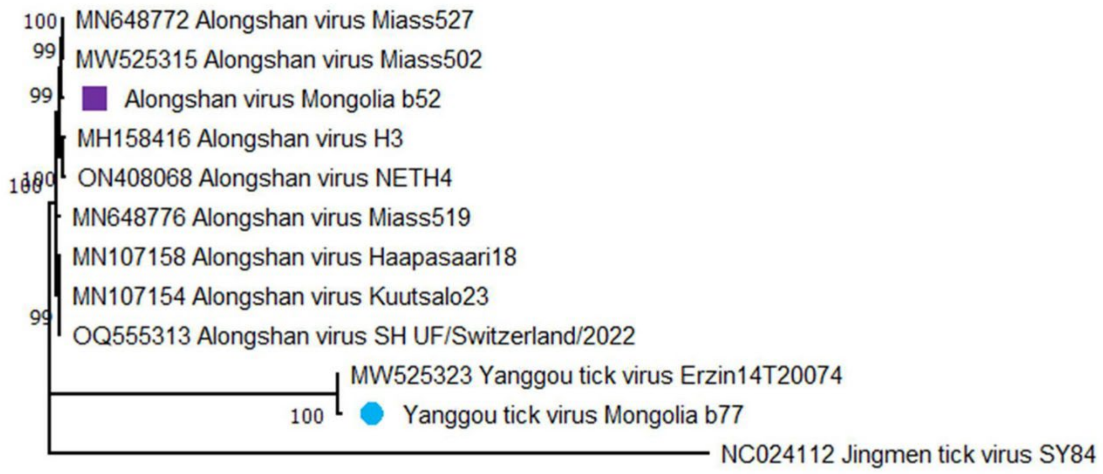

Appendix 1 Figure 5. Maximum-likelihood tree of the Alongshan virus and Yanggou tick virus VP1a-1b sequences (segment 2; 1,080 nucleotides), constructed using Tamura-Nei model, gamma distributed with invariant sites (G+I) for 500 replications. Sequences obtained in the study are marked (purple square and blue octagon) and indicated with sample identifiers. Virus strains are indicated by GenBank accession number, name and isolate identifier. Jingmen tick virus strain SY84 was included as an outgroup.

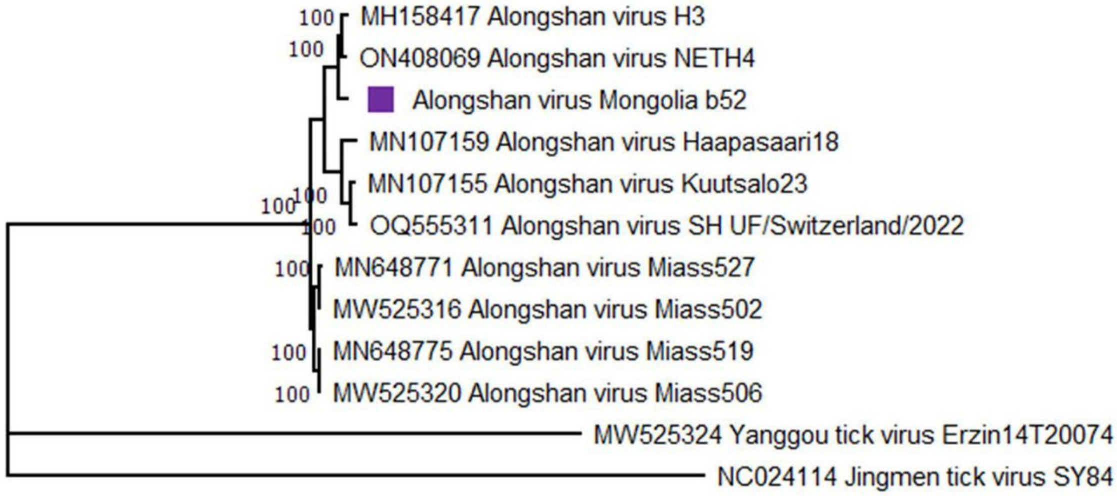

Appendix 1 Figure 6. Maximum-likelihood tree of the Alongshan virus NS3-like protein sequences (segment 3; 1,482 nucleotides), constructed using Tamura-Nei model, gamma distributed with invariant sites (G+I) for 500 replications. Sequence obtained in the study is marked (purple square) and indicated with sample identifiers. Virus strains are indicated by GenBank accession number, name and isolate identifier. Jingmen tick virus strain SY84 was included as an outgroup.

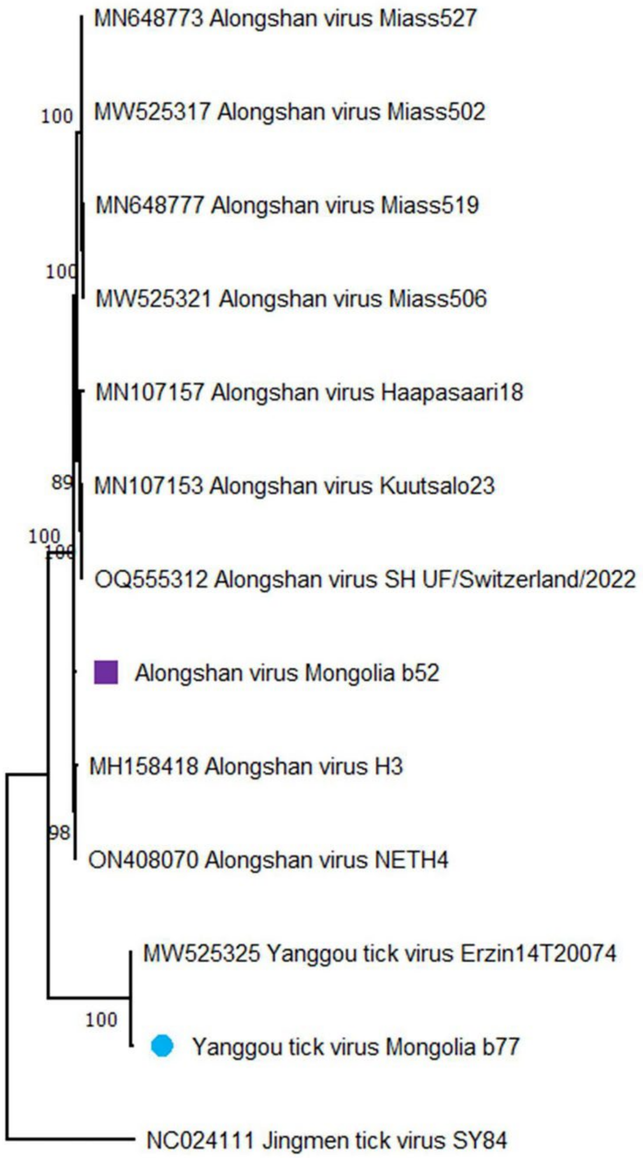

Appendix 1 Figure 7. Maximum-likelihood likelihood tree of the Alongshan virus and Yanggou tick virus VP2-3 sequences (segment 4; 1,080 nucleotides), constructed using Tamura-Nei model, gamma distributed with invariant sites (G+I) for 500 replications. Sequences obtained in the study are marked (purple square and blue octagon) and indicated with sample identifiers. Virus strains are indicated by GenBank accession number, name and isolate identifier. Jingmen tick virus strain SY84 was included as an outgroup.
